# Motor Control of Landing from a Jump in Simulated Hypergravity

**DOI:** 10.1371/journal.pone.0141574

**Published:** 2015-10-27

**Authors:** Clément N. Gambelli, Daniel Theisen, Patrick A. Willems, Bénédicte Schepens

**Affiliations:** 1 Laboratory of Physiology and Biomechanics of Locomotion, Institute of Neuroscience (IoNS), Université catholique de Louvain (UCL), Louvain-la-Neuve, Belgium; 2 Sports Medicine Research Laboratory, Luxembourg Institute of Health, Luxembourg, Grand Duchy of Luxembourg; Scientific Institute Foundation Santa Lucia, ITALY

## Abstract

On Earth, when landing from a counter-movement jump, muscles contract before touchdown to anticipate imminent collision with the ground and place the limbs in a proper position. This study assesses how the control of landing is modified when gravity is increased above 1 *g*. Hypergravity was simulated in two different ways: (1) by generating centrifugal forces during turns of an aircraft (*A300*) and (2) by pulling the subject downwards in the laboratory with a Subject Loading System (*SLS*). Eight subjects were asked to perform counter-movement jumps at 1 *g* on Earth and at 3 hypergravity levels (1.2, 1.4 and 1.6 *g*) both in *A300* and with *SLS*. External forces applied to the body, movements of the lower limb segments and muscular activity of 6 lower limb muscles were recorded. Our results show that both in *A300* and with *SLS*, as in 1 *g*: (1) the anticipation phase is present; (2) during the loading phase (from touchdown until the peak of vertical ground reaction force), lower limb muscles act like a stiff spring, whereas during the second part (from the peak of vertical ground reaction force until the return to the standing position), they act like a compliant spring associated with a damper. (3) With increasing gravity, the preparatory adjustments and the loading phase are modified whereas the second part does not change drastically. (4) The modifications are similar in *A300* and with *SLS*, however the effect of hypergravity is accentuated in *A300*, probably due to altered sensory inputs. This observation suggests that otolithic information plays an important role in the control of the landing from a jump.

## Introduction

Over millions of years, living organisms evolved to cope with Earth’s gravitational environment. Life under 1 *g* is especially challenging for beings practicing bipedal locomotion, such as *Homo sapiens*. For example, fluid flow in the human body is strongly influenced by gravity, and orthostatic regulation is one of the main functions of the autonomic nervous system. During daily living activities, the nervous system must constantly generate appropriate motor commands to ensure postural control and limb dynamics when the organism is submitted to external constraints, including the omnipresent gravity field [1].

During landing from a jump, the lower limb muscles are activated to decelerate the downward motion of the body and dissipate the kinetic energy generated during the fall. A very simple task, such as landing from a vertical jump, involves a predictive behavior to place the limbs in a proper position and generate a muscular force to cope with the forthcoming impact forces [2, 3]. Sensory information from the visual, vestibular and proprioceptive systems contribute to this predictive behavior. Indeed, the deprivation of visual and vestibular information in cats prevented them from correctly positioning their limbs and land on their paws [4].

Furthermore, after touchdown, the strategy of movement will depend on the forthcoming motor task (*e*.*g*. landing followed or not by consecutive jump) and on the time-history and magnitude of the impact forces. When the subject is asked to execute repetitive jumps, the energy lost during landing may be partly stored into the muscle-tendon units to be re-used during the following push-off. This situation can be compared to a mass mounted on a spring bouncing on the ground [5]. However, when the subject is instructed to stand still after landing, the energy lost during the negative work phase must be dissipated to avoid rebounding on the ground. This last behavior does not correspond to a spring-mass bouncing on the ground, but rather to a mass mounted on a damper that dissipates the energy of the jump [6].

When the height of the jump is increased, the vertical velocity of the center of mass (*COM*) at touchdown is greater, which in turn, induces higher impact forces during landing [7–13]. Adaptations to these increased loads involve increased range of motion and muscular negative work during landing, especially at the level of the knee and the hip [10, 13]. Since the acceleration of gravity on Earth varies less than 0.4% over the surface of the globe, humans only experience to fall at 1 *g*. Consequently, the gravitational acceleration is presumably perceived by the Central Nervous System (*CNS*) as a constant parameter to predict the instant of touchdown and the associated vertical velocity of the *COM*. Increasing gravity may affect those predictions, and in turn, the preparatory adjustments and the subsequent biomechanics of landing.

Hypergravity can be simulated by generating centrifugal forces in an aircraft that turns in circles. The resulting centripetal force induces an increased acceleration perpendicular to the floor of the plane, which is felt by the subject as an increased "downward" acceleration. In this way, the whole gravity field seems to be enhanced and the greater pseudo-gravitational constraint is applied to the whole body and generates unusual otolithic signals. When analyzing pointing movements in such an enhanced gravity field, Crevecoeur et al. [1] suggest that the *CNS* takes into account the effect of increased gravity acting on the upper limbs and takes advantage of the dynamic interaction between the body and the environment. In such an environment, the gravitational receptor sensory signals from the otolithic organs are altered, while the semi-circular canal cues are presumably unaffected [14].

Hypergravity can also be simulated into the laboratory by using a Subject Loading System (*SLS*) generating a force pulling the subject downward by means of a harness [15]. In this case, the downward acceleration of the *COM* is increased but the limbs and the otolith organs are still submitted to 1 *g*.

So far most studies investigating the biomechanics of landing from a jump have either used added mass [16, 17] or have manipulated jump/drop height [7–13, 18, 19]. In these cases, the downward acceleration remains equal to 1 *g*. Only Avela et al. [20] and Kramer et al. [21] have used hypergravity simulators similar to the *SLS* to study drop jumps and reactive hops, respectively.

In this study, hypergravity is simulated in two ways: first during turns when flying in the A300 ZERO-G aircraft (*A300*-condition), and second, using a *SLS* [15] generating an pull-down force applied to the trunk (*SLS*-condition). The first aim of this study is to explore the influence of increased gravity on the motor control of landing from a counter-movement jump (*CMJ*) without rebounding. Therefore, the biomechanics of landing at 1.2 *g*, 1.4 *g* and 1.6 *g* are compared with landing at 1 *g*. The second objective is to confront the landing strategies in the *A300-* and in the *SLS*-conditions. We hypothesize that the way hypergravity is simulated will modify the landing pattern, given the different sensory signals provided to the *CNS* in the two situations. A mechanical model is also developed to describe the biomechanics of landing without rebound at different hypergravity levels.

## Materials and Methods

### Subjects and experimental procedures

Eight healthy subjects (Table 1) participated to the study. Subjects were physically active and did not regularly practice sports involving jump-landing tasks. Each participant passed a class II medical examination prior to their enrolment. Experiments were performed according to the principles of the Declaration of Helsinki and were previously approved by the local ethics committee (“Commission d’Éthique Biomédicale Hospitalo-Facultaire de l’Université catholique de Louvain”, (2011/15JUI/322, Belgian Registration Number: B403201111769). Subjects provided their written informed consent prior to participating in the study, following a procedure sanctioned by the ethics committee.

**Table 1:** Population characteristics, number of trials per experimental condition and number of trials per experimental condition where kinematics data were available (in parenthesis) for each subject.

Subject	1 (♀)	2 (♂)	3 (♀)	4 (♀)	5 (♂)	6 (♂)	7 (♂)	8 (♂)
Age (yo)	25	23	45	23	23	42	24	59
Height (m)	1.62	1.72	1.69	1.65	1.69	1.71	1.76	1.72
Weight (kg)	50.0	64.0	60.0	54.0	63.0	75.5	66.0	73.0
Experimentalconditions	Number of trials analyzed							
1 *g*	6 (6)	6 (6)	6 (6)	6 (6)	6 (6)	6 (6)	6 (6)	6 (6)
***A300***								
1.2 *g*	6 (4)	6 (5)	6 (5)	6 (4)	6 (6)	6 (6)	6 (6)	6 (6)
1.4 *g*	6 (3)	6 (5)	5 (5)	6 (5)	6 (6)	6 (6)	6 (4)	6 (0)
1.6 *g*	4 (2)	6 (5)	3 (3)	4 (2)	4 (4)	6 (6)	6 (3)	6 (6)
***SLS***								
1.2 *g*	6 (6)	6 (6)	6 (6)	6 (6)	6 (6)	6 (6)	6 (6)	6 (6)
1.4 *g*	6 (6)	6 (6)	6 (6)	6 (6)	6 (6)	6 (6)	6 (6)	6 (6)
1.6 *g*	6 (6)	6 (6)	6 (6)	6 (6)	6 (6)	6 (6)	6 (6)	6 (6)

Each subject underwent two experimental sessions. During the first session, hypergravity was simulated by a Subject Loading System (*SLS*-condition). During the second session, hypergravity was simulated during turns of an aircraft (*A300*-condition). During both sessions, participants were instructed to start from a standing position, to perform a *CMJ* and to land without rebounding (*i*.*e*. after touchdown, both feet had to stay in contact with the ground until the subject returns to its initial standing position). Subjects were asked to push and land on both feet, to maintain their hands on their hips and keep the gaze horizontal. A trial was considered non-valid if one of these instructions were not fulfilled. No directives were given about the speed of execution of the movement, the height of the jump and the style of landing. After each *CMJ*, the subjects were asked to stand immobile for a period of ~3 s. Then the sequence was repeated until the end of the recording period.

### Simulation of hypergravity

#### *A300*-gravitational environment

The *A300-*sessions were performed in the Airbus A300 ZERO-G aircraft owned by NOVESPACE and based in Bordeaux (France). These sessions took place during the 55^th^ and 56^th^ ESA Parabolic Flight Campaigns organized by the European Space Agency (ESA). Each campaign consisted of three flights; each flight started with 31 parabolic maneuvers that generated sequences of 20 s of hypergravity (1.8 *g*), followed by about 22 s of weightlessness before another period of 20 s of hypergravity at 1.8 *g*. Our experiments took place during turns following the parabolic maneuvers. Each turn of the aircraft lasted ∼50 s, and generated centrifugal forces to simulate hypergravity. For safety reasons required by the flight plan, hypergravity levels were progressively increased: starting with 1.2 *g* during the first turn, then 1.4 *g* during the next turn and finally 1.6 *g*. One or two subjects participated in each flight. Each subject underwent one turn per gravity level. During each turn, the subject performed 6–10 *CMJ*s. Each turn was interspersed by a period of 5 minutes at 1 *g* during which subjects could rest. Note that during the *A300*-session, *CMJ*s at 1 *g* were not performed.

Three accelerometers (DS-Europe, Milan, cut off frequency of 2.5 Hz) placed at the bottom of the experimental set-up measured the three components of the acceleration relative to the reference frame of the aircraft: the mean values of the acceleration along the axis perpendicular to the floor of the plane (*a*_z-A300_) were respectively 1.21±0.01 *g* (mean ±S.D.), 1.41 ±0.01 *g* and 1.61 ±0.01 *g*.

#### *SLS*-gravitational environment

**Fig 1:**
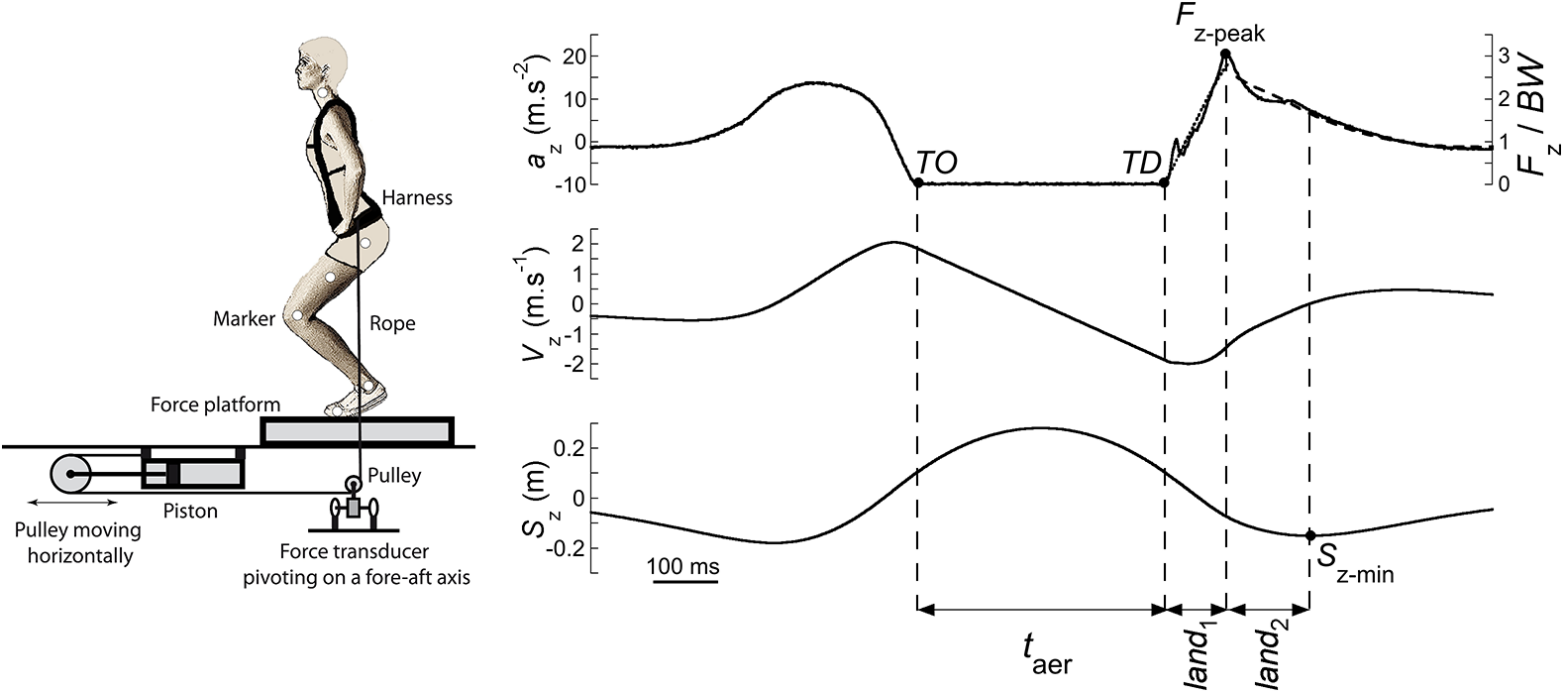
Experimental set-up (left) and typical trace in 1 *g* (right). **Left:** scheme of the experimental set-up in the laboratory to simulate hypergravity. The additional pull-down force simulating hypergravity was generated by two pneumatic pistons placed horizontally on each side of the subject under the ground level. The force generated by each piston was transmitted to the harness by a rope, which passed through two pulleys. A first pulley, moving horizontally, doubled the movement of the harness as compared to the piston. A second pulley changed the direction of the force from horizontal to vertical. A force transducer placed at the level of this last pulley measured the tension in the rope and a force-plate measured the ground reaction forces under the feet (for more details see Methods). **Right:** typical trace of one subject (60 kg, 1.69 m, 46 yo) in 1 *g* in the laboratory: the vertical component of the ground reaction force (*F*_z_ normalized in *BW*), the vertical acceleration (*a*_z_), velocity (*V*_z_) and displacement (*S*_z_) of the *COM* are expressed as a function of time, from 500 ms before take-off (*TO*) until 500 ms after touchdown (*TD*). The jump is divided into sub-periods: the instant of *TO* and the instant of *TD* delimit the aerial phase (*t*_aer_). The *land*_1_ is the period between *TD* and the moment at which the vertical force reaches its peak (*F*_z-peak_); *land*_2_ is the period between the time of *F*_z-peak_ and the moment at which the *COM* reaches its lowest vertical position (*S*_z-min_). The dotted line on the *a*_z_-time curve during *land*_1_ represents the function computed from a spring-mass model. The black interrupted line after *land*_1_ represents the function computed from a damped harmonic oscillator model (see Methods).

The *SLS*-sessions were performed in the laboratory, a few weeks prior the parabolic flight campaign. The Subject Loading System, similar to the one described by Gosseye et al. [15], consisted of two pneumatic pistons, generating a pull-down force transmitted to a harness on either side of the subject (Fig 1, left). The harness was a copy of the one used in the MIR station by the Russian Federal Space Agency (ROSCOSMOS). It was composed of a large pelvic belt and adjustable shoulder straps, distributing the pull-down force between the hips and the shoulders. The harness did not constrain any movements of the lower and upper limbs. Each rope passed through a pulley mounted on a ball bearing below the landing surface; the pulleys were equipped with a force transducer measuring the vertical component of pull-down force in the rope (*F*_t_). Slight variations in *F*_t_ were observed, due to friction and inertia in the pistons, as well as pressure drops in the pneumatic system: *F*_t_ was greater than the expected value when the subject moved upward (+0.08±0.02 *g* in 1.2 *g-SLS*, +0.12±0.02 *g* in 1.4 *g-SLS* and +0.14±0.03 *g* in 1.6 *g-SLS*) and smaller when the subject moved downwards (-0.01±0.01 *g* in 1.2 *g-SLS*, -0.02±0.02 *g* in 1.4 *g-SLS* and -0.03±0.02 *g* in 1.6 *g-SLS*). The mean value of *F*_t_ calculated from the beginning until the end of the jumps performed was 0.22±0.01 *BW* (mean±S.D.) for the 1.2 *g-SLS*, 0.43±0.02 *BW* for the 1.4 *g-SLS* and 0.63±0.01 *BW* for the 1.6 *g-SLS*, where *BW* is the body weight on Earth.

Prior to the *SLS*-session, subjects performed several *CMJ*s at 1 *g* (*i*.*e*. without *SLS*) to familiarize with the procedures. Then the following 6 *CMJ*s were recorded as reference traces. These were followed by *CMJ*s with the *SLS*. As during the *A300*-sessions, the magnitude of *F*_t_ was set to simulate first 1.2 *g*, then 1.4 *g* and finally 1.6 *g*. For each subject, a minimum of 9 *CMJ*s was recorded at each gravity level. A 1 minute rest period was provided every 3 *CMJ*s.

### Experimental set-up

#### Measurement of the vertical ground reaction force

The vertical and fore-aft component of the ground reaction force were measured by means of a 0.6 x 0.4 m platform, instrumented with strain gauges [22], during *SLS*-sessions and during the 56^th^ ESA Parabolic Flight Campaign, or with piezoelectric sensors (KISTLER), during the 55^h^ ESA Parabolic Flight Campaign. In this study, only the vertical component of the force (*F*_z_) was used for analysis.

#### Measurement of the kinematics of the lower limb segments

The movements of the left lower limb segments were measured in the sagittal plane by means of a high-speed video camera (BASLER piA640-210, resolution 486 x 646 pixels). The camera was fixed 3 m to the side of the force platform perpendicular to the sagittal plane. The camera field encompassed about 1.7 x 2.4 m, allowing the measurement of the body segments during the whole *CMJ*. Images were sampled at a rate of 100 Hz. Reflective markers were taped on the skin on the left side of the subject, at the level of the chin-neck intercept, the greater trochanter, the upper part of the lateral side of the thigh, the external femoral condyle, the lateral malleolus, and the fifth metatarsal phalangeal joint (Fig 1, left). This procedure assumed that the movements of both lower limbs were identical.

#### Measurement of the electromyographic activity

The muscular activity of six muscles of the left lower limb was recorded by means of electromyography (*EMG*) using bipolar electrodes (IMMED E111, diameter: 30 mm, inter-electrode distance: 20 mm). This procedure assumed that the muscular activity of both lower limbs were identical. The skin was cleaned with ether and alcohol before applying the electrodes. Absence of movement artifacts was verified by tapping the electrodes and by shaking the wires. Electrodes were placed according to the SENIAM recommendations (http://www.seniam.org) on the tibialis anterior (*TA*), peroneus brevis (*PB*), gastrocnemius lateralis (*GL*), soleus (*Sol*), vastus lateralis (*VL*) and biceps femoris (*BF*) muscles. The electrodes were connected to a MyoSystem 1400L (Noraxon, USA) (gain: 60 dB; input impedance: 100 MΩ; common mode rejection ratio at 50–60 Hz: -100 dB; band-pass: 10–500 Hz).

### Acquisition and signal processing

The force and *EMG* signals were digitized with a 16-bit A/D convertor (NI PCI 6229, National Instruments, Austin, TX, USA) at a sampling frequency of 1000 Hz. The A/D convertor was synchronized with the camera by means of a trigger signal. Acquisition and signal processing were performed using custom software (LABVIEW 2010, National Instruments, Austin, TX, USA).

#### Computation of the vertical acceleration, velocity and displacement of the *COM*

The vertical acceleration, velocity and displacement of the *COM* were computed from the force recordings [22, 23]. Each jump started with the subject standing still prior to the initiation of the *CMJ*, and ended when the subject returned to his/her initial upright position; *i*.*e*. when the vertical position of the marker on the chin-neck intercept at the end of the *CMJ* was within ± 0.03 m of its position at the beginning.

In 1 *g* in the laboratory, the vertical acceleration of the *COM* (*a*_z_) was computed as:

$$a_{\text{z}}\;=\;{(F}_{\text{z}}-BW)/m$$

where *m* is the body mass and *BW* is the body weight on Earth. In *A300*-condition, *a*_z_ was computed as:

$$a_{\text{z}}\;=\;\left(F_{\text{z}}-a_{\text{z}-A300}m\right)/m,$$

and in *SLS*-condition, *a*_z_ was computed as:

$$a_{\text{z}}\;=\;\left(F_{\text{z}}-BW-F_{\text{t}}\right)/m.$$

The time-integration of *a*_z_ from the beginning to the end of the jump resulted in the vertical velocity of the *COM* (*V*_z_). Since the average vertical velocity was nil over the entire jump, the integration constant was set to zero. The time-integration of *V*_z_ yielded the vertical displacement (*S*_z_) of the *COM* (*S*_z_ = 0 corresponded to the standing position).

#### Time-division of the *CMJ*

The right part of Fig 1 presents the *a*_z_, *F*_z_/*BW*, *V*_z_ and *S*_z_-time curves during a *CMJ* in the 1 *g*-condition (*i*.*e*. in the laboratory, without harness). Take-off (*TO*) was determined as the last point where *F*_z_/*BW* > 0.01 and touchdown (*TD*) as the last point where *F*_z_/*BW* < 0.01.

Before *TO*, the subject generated an impulse to jump off the ground and the momentum of the *COM* increased. The aerial phase (*t*_aer_) was defined as the period between *TO* and *TD*. Shortly after *TD*, the momentum of the *COM* decreased, this negative work phase was divided into two parts:

- *land*_1_ (Fig 1), the period between *TD* and the moment at which the *F*_z_ reached its maximum (*F*_z-peak_); in this phase, *F*_z_ increased while *S*_z_ decreased, and
- *land*_2_ (Fig 1), the period between *F*_z-peak_ and the moment at which the *COM* reached its lowest point (*S*_z-min_; *i*.*e*. when *V*_z_ is nil). In this phase, *F*_z_ decreased while *S*_z_ still decreased.

The sum *land*_1_ + *land*_2_ is referred to as the *landing* period. After *land*_2_, *S*_z_ increased until the *COM* reached its initial standing position.

#### Modeling landing without rebound

To understand the influence of hypergravity on the measured variables (kinetic, kinematics and *EMG*), a simple model was designed using the time-division presented above. To our knowledge, only few studies implemented models that took into account energy dissipation during landing. As suggested by Dyhre-Poulsen et al. [6], muscle behavior should change during landing from a spring to a damper to dissipate the energy and to avoid rebound after *TD*. Therefore, we divided the landing phase into two parts: during *land*_1_, muscles were acting like a spring and during *land*_2_ until the end of the jump, muscles were acting like a damped harmonic oscillator.

During *land*_1_, the mass-specific overall stiffness (*k*_1_) of the spring-mass system was calculated as the slope of the *a*_z_ *-S*_z_ curve, computed by a linear regression. During the second part of landing, the mass-specific overall stiffness (*k*_2_) and the mass-specific damping coefficient (*c*_2_) of the system were estimated using a regression model. At each instant *i*, the general equation of a damped harmonic oscillator was given by:

$$a_{\text{z}}\left(i\right)\;=\;k_{2}S_{\text{z}}\left(i\right)+c_{2}V_{\text{z}}\left(i\right)+intercept,$$

where *a*_z_(*i*), *S*_z_(*i*) and *V*_z_(*i*) were the experimental data at the instant *i*. In this way, *n* equations were produced, where *n* was the number of samples of the second part of the landing. This set of equations resulted in an over-constrained system from which we searched the least square solution, to obtain *k*_2_ and *c*_2_.

#### Kinematic signal processing

Coordinates of the reflectors in the sagittal plane were measured each frame using the Lynxzone software (ARSALIS, Belgium). A spline function was fitted through the experimental data to smoothen the signal and to interpolate existing points to obtain a 1000 Hz signal. The joint angles were calculated on each frame [12] and the range of motion during *land*_1_ (*RoM*_1_) and *land*_2_ (*RoM*_2_) was calculated as the difference between the maximal and the minimal joint angle measured during each of these phases. When the marker on the left greater trochanter was hidden by the harness, its position was calculated from the orientation of the segment defined by the marker on the femoral condyle and the marker on the lateral side of the thigh (Fig 1, left) and from the distance between the reflective markers measured with an anthropometer prior to the experiment. In the *A300*, due to technical issues kinematics could not be recorded during some trials (Table 1).

#### *EMG* signal processing

The *EMG* signals were filtered using a fourth-order zero-phase-shift Butterworth digital filter band-pass (20–500 Hz) and then rectified. For each subject in each experimental condition, these raw rectified *EMGs* were synchronized relative to *TD* and averaged point by point (subject's mean trace). Then in each experimental condition, the grand mean trace of all subjects was obtained by averaging the subject's mean trace over periods of 5 ms.

In the 1 *g*-condition, an *EMG* 'silent phase' was generally present during the aerial phase, between the end of the 'push-off contraction' and the onset of the 'pre-landing contraction'. The pre-landing activation was defined as the period between the time at which muscle initiate contraction during the aerial phase and *TD*. It was determined jump by jump using the method of Santello and McDonagh [11]. In hypergravity, since the aerial phase became shorter, the 'silent phase' tended to disappear, which made the detection of the onset of the pre-landing activity unreliable. Therefore this variable was not measured in the *SLS* and *A300*-conditions.

### Data reduction and statistics

When jumping in hypergravity (both in *A300-* and *SLS*-conditions), we observed *a posteriori* an adaptation during the three first jumps. Since our study is intended to analyze the modifications of the control of jumping with increased gravity in a 'steady state' condition (and not during the transition between one gravity level and another), the first 3 trials of each experimental condition were systematically discarded and kept for further analysis. Only the subsequent trials were analyzed: 6 trials were analyzed in the *SLS*-condition whereas only 3–6 trials could be analyzed in the *A300*-condition (Table 1). In order to check if the trials analyzed were realized in steady state, a linear regression on the *jump height vs trial number* was performed. The regression analysis revealed that the slope of the linear fit was never significantly different from zero for each subject in each experimental condition.

The statistical analysis was designed to assess the effect of the gravity level (1, 1.2, 1.4 and 1.6 *g*) of the gravitational environment (*A300* or *SLS*) and of the interaction between gravity level and gravitational environment. Since no *CMJ* could be recorded at 1 *g* during the *A300*-session, the 1 *g*-condition performed in the laboratory was taken as the reference both for the *A300-* and *SLS-*conditions. As the landing strategy may differ from one subject to another, a within-subject Analysis of Variance (ANOVA) was selected. Specifically, a two-level linear mixed model ANOVA with Bonferroni post-hoc tests was applied. Gravitational environment and gravity level were set as fixed effects, and the subject was set as a random effect on a total of 374 trials (48 trials in 1 *g-*condition, duplicated, plus 134 trials in *A300-* and 144 in *SLS*-condition). The normality of the residuals was checked visually with QQ-plots. In addition, normality of the residuals was not assumed if asymmetry was superior than 1.5 (or inferior than -1.5). Normality of the residuals was not assumed for 3 variables (*land*_2_, *landing*, and *k*_1_). In those cases, a log10 transform was applied. Normality of the residuals was then rechecked and was not assumed for *k*_1_. In this particular case, a reciprocal transform was applied and normality of the ensuing residuals was assumed. The significance level was fixed at *p*<0.05.

The trials of each subject in each experimental condition were averaged. The mean and standard deviation of the ensuing averages were then calculated (grand mean) and are presented in the results and figures.

## Results

### Landing from a *CMJ* in 1 *g*

**Fig 2:**
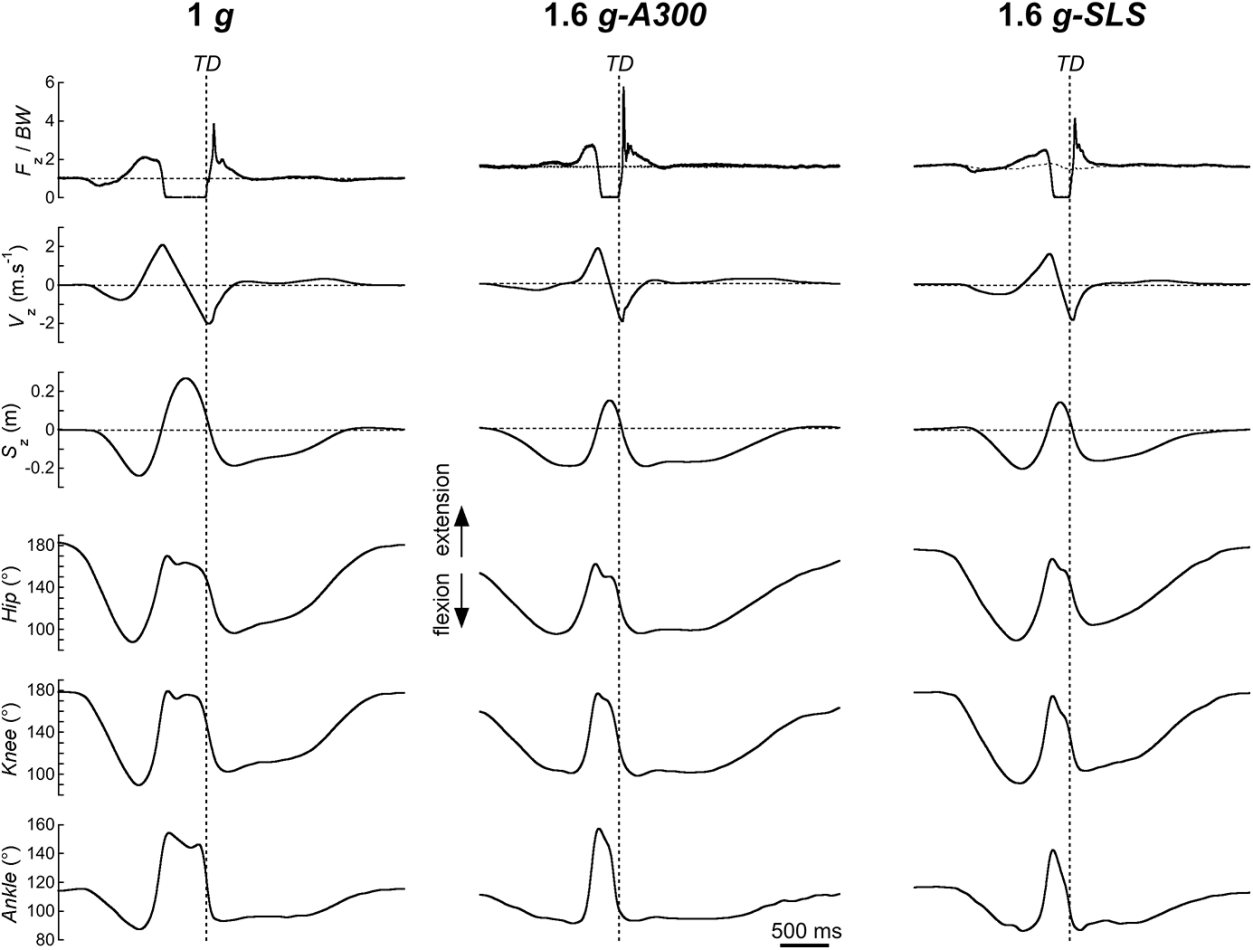
Counter-movement jump in 1 *g* and 1.6 *g*. Typical traces of the kinetics and kinematics as a function of time, during a counter-movement jump (*CMJ*) of a female subject (54 kg, 1.65 m, 23 yo). **Left:** 1 *g*, **middle:** 1.6 *g-A300*, **right:** 1.6 *g-SLS*. Traces start when the subject initiates the *CMJ*, and end when the subject returns to the standing position. **Three top panels**: (from top to bottom): vertical component of the ground reaction force (*F*_z_ normalized in *BW* on Earth), vertical velocity (*V*_z_) and vertical displacement (*S*_z_) of the *COM*. On the *F*_z_/*BW* trace, the horizontal dotted line indicates: *BW* at 1 *g* (left), 1.6 *BW* in the *A300-*condition (middle) and *F*_t_ in the *SLS-*condition (right). **Three bottom panels:** (from top to bottom): angle of the left hip, knee and ankle joints. The vertical dotted lines indicate the instant of touchdown (*TD*).

**Fig 3:**
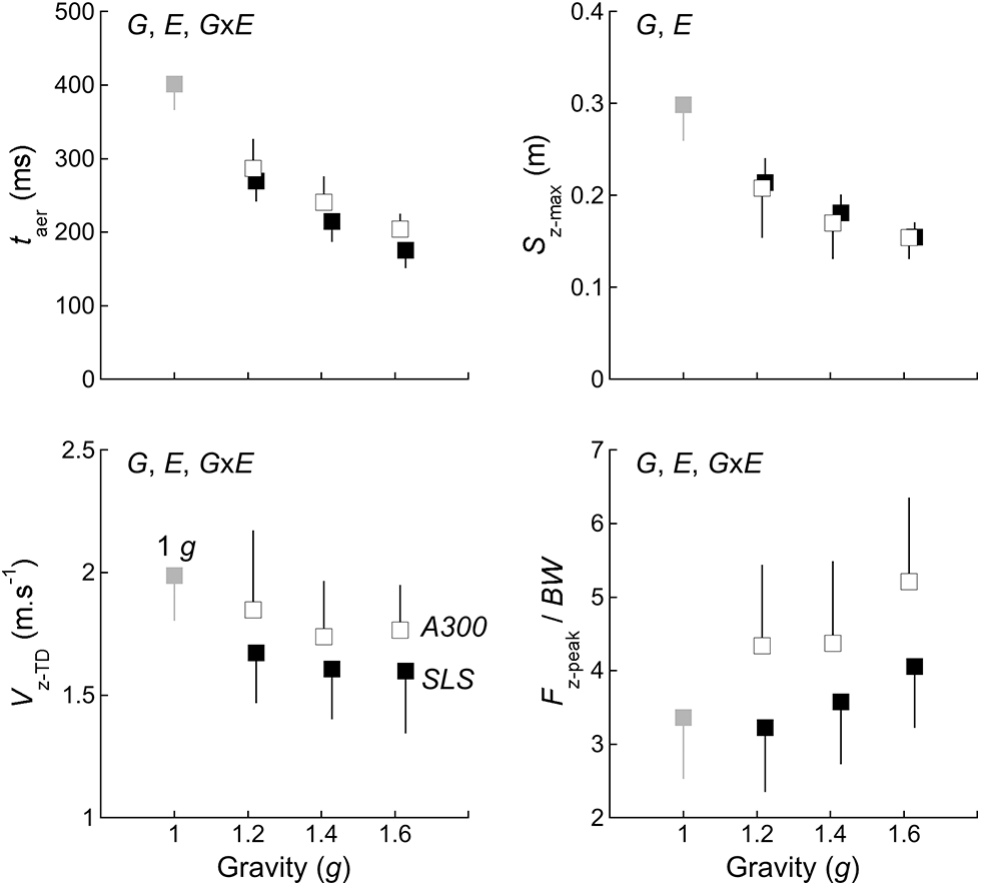
Effect of the experimental conditions on kinetic variables. The aerial time (*t*_aer_), the maximal height of the jump (*S*_z-max_), the vertical velocity of the *COM* at *TD* (*V*_z-TD_) and the maximal vertical ground reaction force normalized by body weight on Earth. (*F*_z-peak_/*BW)* are plotted as a function of the gravity level. Points represent the grand mean of all subjects ± one standard deviation. Grey symbol are for the 1 *g-*condition in the laboratory; black symbol are for the *SLS*-condition and white symbol for the *A300*-condition. In each panel, *G* indicates a significant effect of gravity, *E* the effect of the experimental condition (*A300* or *SLS*) and *G*x*E* the interaction between gravity and experimental condition (*p*<0.05).

**Fig 4:**
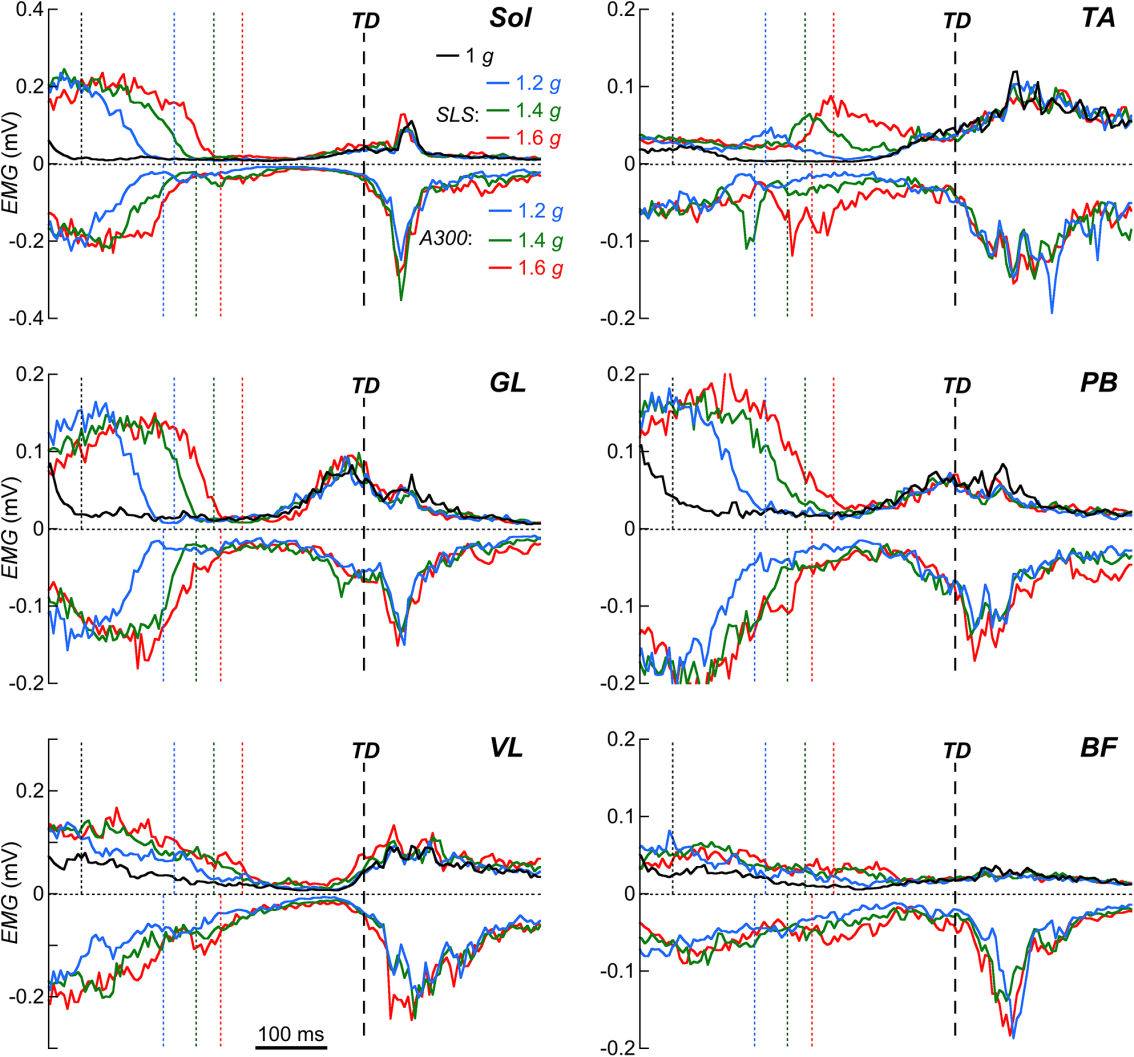
Effect of the experimental conditions on the *EMG*–pattern. *EMG*-patterns of soleus (*Sol*), tibialis anterior (*TA*), gastrocnemius lateralis (*GL*), peroneus brevis (*PB*), vastus lateralis (*VL*) and biceps femoris (*BF*) muscles are presented from 450 ms before *TD* to 250 ms after *TD*. Positive traces are for the 1 *g* and the *SLS*-conditions and negative traces for the *A300*-condition. Black line is for 1 *g*; blue lines are for 1.2 *g*, green for 1.4 *g* and red for 1.6 *g*. Traces are the grand mean of the eight subjects averaged over periods of 5 ms and are synchronized to *TD* (vertical interrupted line). Dotted vertical colored lines correspond to the *TO* (same color than the curves) in each condition.

The left column of Fig 2 presents typical traces of a *CMJ* performed at 1 *g*. At first, the subject stands still in an upright position. During the impulse, the subject squats and extends the lower limbs. The vertical velocity of the *COM* (*V*_z_) reaches a maximum slightly before *TO*. On the average, the aerial phase (*t*_aer_) lasts 402±34 ms (grey symbol in Fig 3). During *t*_aer_, *V*_z_ decreases and increases linearly with a slope of -1 *g* (Fig 2) and *S*_z_ reaches a maximum of 0.30±0.04 m (Fig 3). The hip, knee and ankle start to flex before *TD* (Fig 2). The *PB*, *GL* and *TA* muscles are first activated about 100 ms before *TD* (respectively 106±27 ms, 104±25 ms and 82±23 ms); then the *BF* and *Sol* muscles are activated about 70 ms before *TD* (respectively 72±32 ms and 67±23 ms) and at last, the *VL* muscle is activated about 20 ms before *TD* (24±14 ms) (Fig 4).

**Fig 5:**
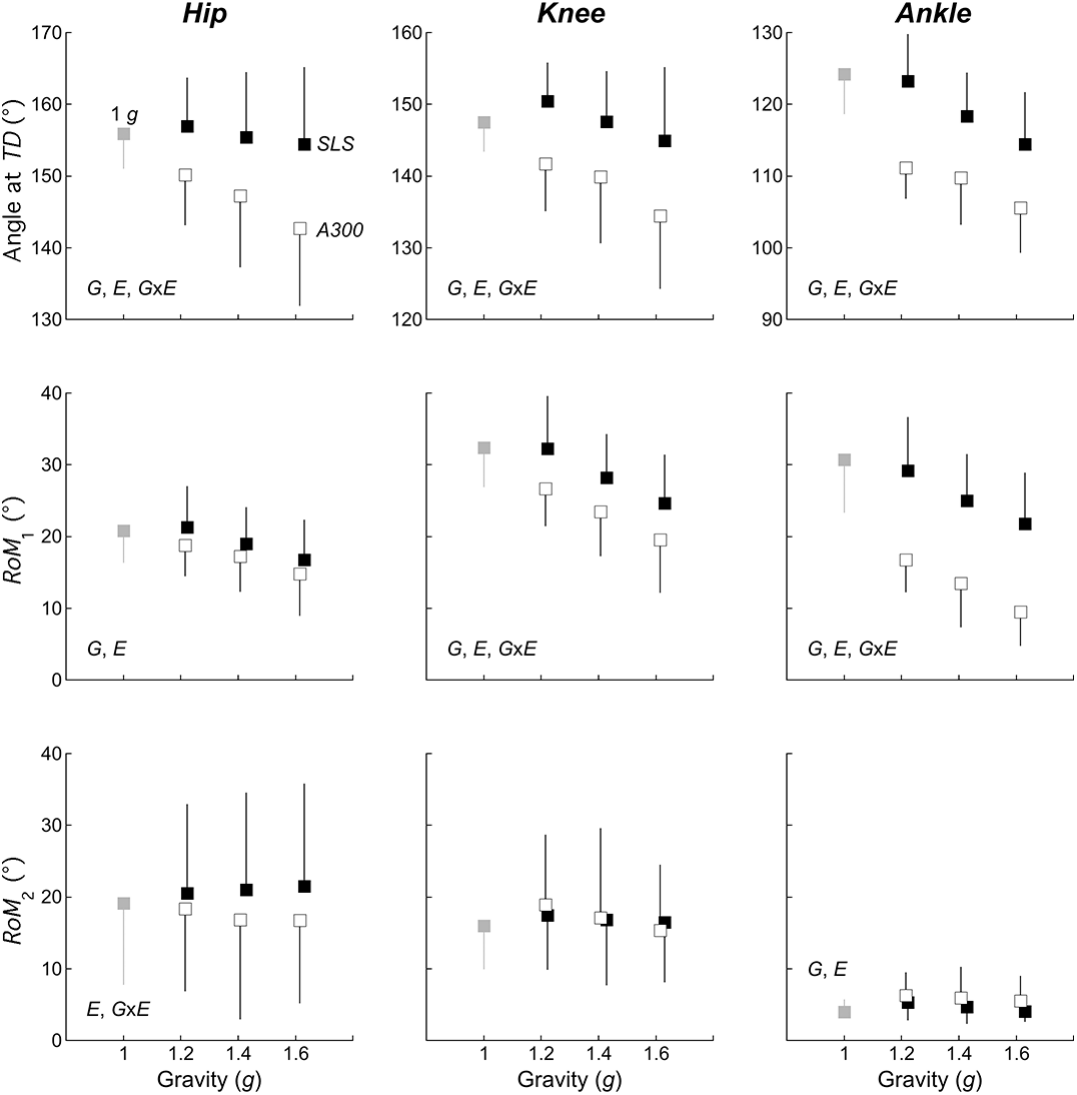
Effect of the experimental conditions on the kinematics of the lower limb joints. **Top panels:** Angle of the hip, knee and ankle joint at *TD*, **Middle panels:** range of motion of the lower limb joints during *land*_1_ (*RoM*_1_), **Bottom panels,** from left to right: range of motion of the lower limb joints during *land*_2_ (*RoM*_2_). Other indications as in Fig 3.

At *TD*, the vertical velocity of the *COM* is equal to 2.0±0.2 m s^-1^ (*V*_z-TD_ in Fig 3) and the *COM* is ~0.10 m higher than during standing (Fig 2). The angle of the hip, knee, and ankle are 156±5°, 148±4° and 124±6°, respectively (Fig 5). All subjects first touched the ground with the forefoot and then the heel touched the ground. After *TD*, *V*_z_ still increases during ~30 ms (Fig 2) and *F*_*z*_ reaches its maximum *F*_z-peak_ = 3.4±0.8 *BW* (Fig 3) on the average 86±18 ms after *TD* (see duration of *land*_1_ in Table 2). During *land*_1_, *S*_z_ decreases by 0.16±0.03 m (Table 2) and the range of motion of the hip, knee, and ankle (*RoM*_1_) is respectively 21±4°, 32±5° and 31±7° (Fig 5). After *F*_z-peak_, the lower limb joints are still flexing (Fig 2). During *land*_2_, *S*_z_ decreases by 0.11±0.04 m (Table 2) and the range of motion of the hip, knee, and ankle (*RoM*_2_) is respectively 19±11°, 16±6° and 4±2° (Fig 5). Note that most of the vertical displacement of the *COM* and most of the range of motion of the lower limb joints take place during *land*_1_. At the end of landing phase, the subject returns to his initial standing position.

**Table 2:** Effect of the experimental conditions on landing periods and on the vertical displacement of the *COM* during *land*_1_ (Δ*S*_z1_), during *land*_2_ (Δ*S*_z2_) and during *landing* (Δ*S*_z_)

Variable	1 *g*	1.2 *g*		1.4 *g*		1.6 *g*	
		*SLS*	*A300*	*SLS*	*A300*	*SLS*	*A300*
***land***_**1**_ **(ms)**	86±18	**87±21**	69±15	**80±18**	66±15	**73±21**	54±16
***land***_**2**_ **(ms)**	183±68	**207±86**	246±95	**239±106**	252±104	**258±123**	240±105
***landing* (ms)**	268±79	**294±99**	315±102	**319±106**	318±113	**331±121**	294±109
Δ***S***_**z1**_ **(m)**	0.16±0.03	**0.15±0.03**	0.13±0.03	**0.13±0.02**	0.12±0.02	**0.12±0.03**	0.10±0.03
Δ***S***_**z2**_ **(m)**	0.11±0.04	**0.11±0.05**	0.15±0.06	**0.12±0.06**	0.15±0.07	**0.11±0.06**	0.14±0.07
Δ***S***_**z**_ **(m)**	0.27±0.06	**0.26±0.07**	0.28±0.07	**0.25±0.07**	0.26±0.08	**0.24±0.06**	0.24±0.08

Figures represent the grand mean ± the standard deviation of all subjects.

### Effect of increased gravity level

The middle and right columns of Fig 2 present typical traces performed at 1.6 *g* in both experimental conditions (*A300* and *SLS*). As compared to 1 *g*, the general pattern of the *CMJ* does not seem drastically modified; however at first glance one can observe that the aerial phase is shorter and that the height of the jump is reduced. Fig 3 shows that when gravity increases, *t*_aer_ is reduced from ~400 ms at 1 *g* to less than ~200 ms at 1.6 *g* and that *S*_z-max_ decreases from ~0.3 m to ~0.15 m. At *TD*, *V*_z-TD_ is reduced, however Bonferroni post-hoc test reveals no significant differences between 1.4 *g* and 1.6 *g* (in Fig 3), and the lower limb joints are more flexed (Fig 5). With increased gravity, *land*_1_ is reduced (Table 2) and *F*_z-peak_ is augmented (Fig 3). During *land*_1_, the vertical displacement of the *COM* (Δ*S*_z1_ in Table 2) and the *RoM*_1_ of the lower limb joints (Fig 5) are reduced with increased gravity. During *land*_2_, Δ*S*_z2_ increases with gravity (Table 2), however Bonferroni post-hoc test reveals that only Δ*S*_z2_ at 1 *g* is different from the other gravity levels. Except for the ankle, *RoM*_2_ is not modified by hypergravity (Fig 5). Detailed *F*, *p* and partial eta^2^ values in increased gravity (*G*), in the two experimental conditions (*E*), and interaction between *G* and *E* are presented in Table 3.

**Table 3:** Statistical analysis: effect of the gravity *G* (1 *g*, 1.2 *g*, 1.4 *g* and 1.6 *g*), of the experimental condition *E* (*SLS* or *A300*), and of the interaction between *G* and *E*.

Variable	*G* (*F* / *p* / partial eta^2^ values)	*E* (*F* / *p* / partial eta^2^ values)	*G* x *E* (*F* / *p* / partial eta^2^ values)
Kinetics			
*F*(DoF effect, DoF error)	*F*(3,359)	*F*(1,359)	*F*(3,359)
gravity (*g*)	26215.6 / <0.001 / 0.995	47.3 / <0.001 / 0.116	8.3 / <0.001 / 0.064
*land*_1_ (ms)	59.8 / <0.001 / 0.333	117.2 / <0.001 / 0.246	14.1 / <0.001 / 0.105
*land*_2_ (ms)^$^	17.5 / <0.001 / 0.128	7.4 / 0.007 / 0.020	1.8 / 0.146 / 0.015
*landing* (ms)^$^	9.2 / <0.001 / 0.071	0.0 / 0.991 / <0.001	1.4 / 0.254 / 0.011
*t*_aer_ (ms)	3413.5 / <0.001 / 0.966	137.2 / <0.001 / 0.276	18.2 / <0.001 / 0.132
*S*_z-max_ (m)	694.4 / <0.001 / 0.853	3.9 / 0.048 / 0.011	1.0 / 0.372 / 0.009
*V*_z-TD_ (m.s^-1^)	89.3 / <0.001 / 0.427	57.6 / <0.001 / 0.138	7.0 / <0.001 / 0.055
*F*_z-peak_/*BW*	84.2 / <0.001 / 0.413	180.3 / <0.001 / 0.334	22.4 / <0.001 / 0.157
Δ*S*_z1_ (m)	135.8 / <0.001 / 0.532	51.7 / <0.001 / 0.126	6.5 / <0.001 / 0.051
Δ*S*_z2_ (m)	16.3 / <0.001 / 0.120	79.4 / <0.001 / 0.181	9.1 / <0.001 / 0.070
Δ*S*_z_ (m)	24.2 / <0.001 / 0.168	15.3 / <0.001 / 0.041	1.9 / 0.133 / 0.015
*k*_1_(s^-2^)*	161.4 / <0.001 / 0.574	84.6 / <0.001 / 0.191	11.5 / <0.001 / 0.088
*k*_2_ (s^-2^)	0.7 / 0.562 / 0.006	0.8 / 0.361 / 0.002	0.4 / 0.718 / 0.004
*c*_2_ (s^-1^)	9.9 / <0.001 / 0.077	1.2 / 0.281 / 0.003	0.3 / 0.792 / 0.003
Kinematics			
*F*(DoF effect, DoF error)	*F*(3,332)	*F*(1,332)	*F*(3,332)
Hip angle at *TD* (°)	23.8 / <0.001 / 0.117	110.0 / <0.001 / 0.249	15.5 / <0.001 / 0.123
Knee angle at *TD* (°)	17.4 / <0.001 / 0.137	80.6 / <0.001 /0.196	9.9 / <0.001 / 0.082
Ankle angle at *TD* (°)	51.5 / <0.001 / 0.317	73.6 / <0.001 / 0.179	10.5 / <0.001 / 0.086
Hip *RoM*_1_ (°)	35.3 / <0.001 / 0.242	14.5 / <0.001 / 0.041	2.3 / 0.074 / 0.021
Knee *RoM*_1_ (°)	85.9 / <0.001 / 0.437	58.4 / <0.001 / 0.149	7.7 / <0.001 / 0.065
Ankle *RoM*_1_ (°)	109.9 / <0.001 / 0.498	204.2 / <0.001 / 0.380	25.2 / <0.001 / 0.185
Hip *RoM*_2_ (°)	0.6 / 0.595 / 0.006	19.3 / <0.001 / 0.055	3.1 / 0.028 / 0.027
Knee *RoM*_2_ (°)	2.1 / 0.098 / 0.019	0.1 / 0.774 / <0.001	0.3 / 0.850 / 0.002
Ankle *RoM*_2_ (°)	6.8 / <0.001 / 0.058	8.8 / 0.003 / 0.026	1.2 / 0.301 / 0.011

^$^ indicates that a log10 transform was applied to the variables, * indicates that a reciprocal transform was applied to the variable, as the normality of the residuals was not assumed.

Fig 4 illustrates how the *EMG* time*-*pattern is related to the instant of *TO* and *TD*. In each condition, the *EMG* pattern of each muscle can roughly be superimposed between ~100 ms before and ~100 ms after *TD*. This qualitative assessment suggests that within a session (*A300* or *SLS*) both the time-pattern and the activation of the muscles are similar whatever the gravity level. Both *TA* and *BF* muscles are activated continuously during the aerial phase in hypergravity and the amplitude of this activity seems to increase with increased gravity level.

### Effect of the experimental condition (*A300* vs *SLS*)

In both experimental conditions (*A300* and *SLS*), hypergravity modifies most of the variables measured. However, the magnitude of these changes are not always the same in the two experimental conditions. As compared to the *SLS*, *t*_aer_ is increased in the *A300* while *S*_z-max_ is similar (Fig 3). At *TD*, *V*_z-TD_ is higher in *A300* and the lower limb joints are more flexed (Fig 5). In *A300*, the duration of *land*_1_, Δ*S*_z1_ (Table 3) and *RoM*_1_ (Fig 5) are also reduced, as compared to *SLS*. At the end of *land*_1_, *F*_z-peak_ is higher in *A300*. During *land*_2_, there is an effect of the experimental condition on *RoM*_2_ at the level of the hip and the ankle, but not at the level of the knee (Fig 5). In both *A300* and *SLS*, muscles are active before and after *TD*, however, in *A300* the *EMG* activity after *TD* seems enhanced, as compared to *SLS* (Fig 4).

### Modeling the landing without rebound

In Fig 1, the interrupted lines drawn on the upper trace after *TD* represents the interaction between the *COM* and the landing surface, computed with the model described in the Methods section. In the 1 *g*-condition, the spring-mass model during *land*_1_ shows a good correlation with the experimental data (*r* ^2^ = 0.91±0.08, *n* = 48). Similarly, the damped harmonic oscillator model during *land*_2_ until the end of the jump fits well with the experimental data (*r* ^2^ = 0.89±0.07, *n* = 48). In the *A300*-condition, *r* ^2^ = 0.86±0.11 for the spring-mass model and 0.79±0.13 (*n* = 134) for the damped harmonic oscillator model, while in the *SLS*-condition, *r* ^2^ = 0.92±0.08 (*n* = 144) and 0.85±0.08 (*n* = 144), respectively.

**Fig 6:**
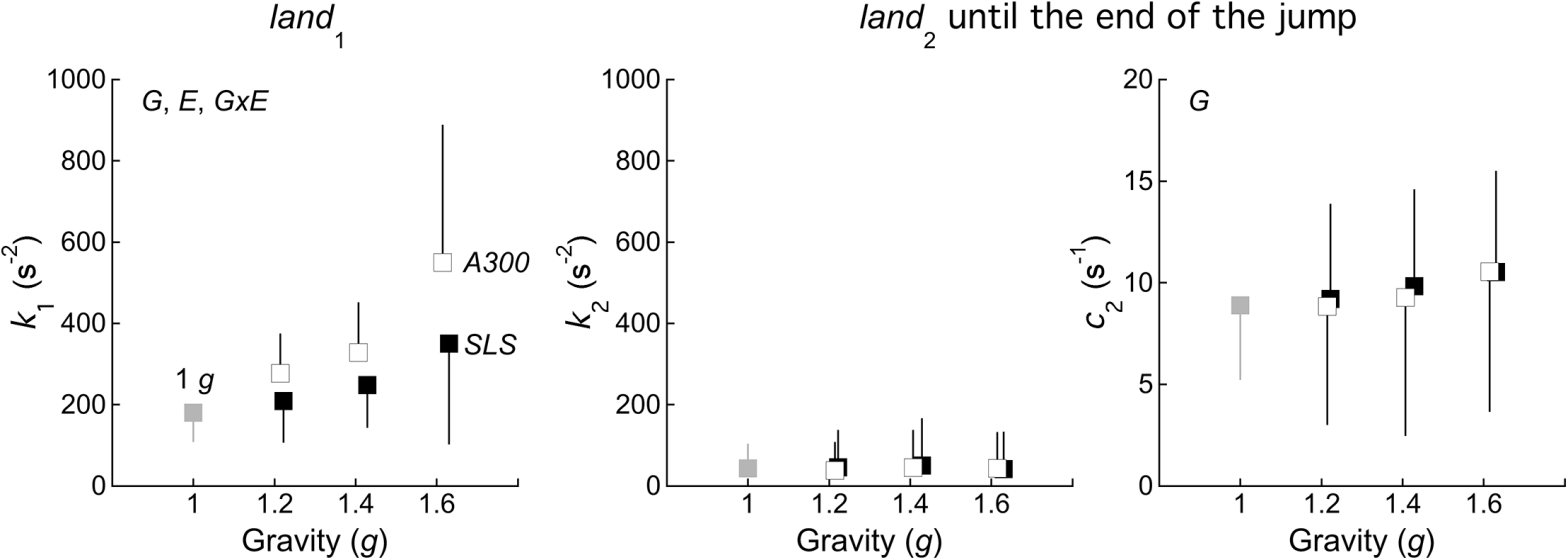
Effect of experimental conditions on the parameters of the biomechanical model of landing. **Left:** overall mass-specific stiffness *k*_1_ generated by the lower limb muscles during *land*_1_. **Middle and right:** overall mass-specific stiffness *k*_2_ and damping coefficient *c*_2_ generated by the lower limb muscles during the second part of landing (*i*.*e*. during and after *land*_2_, until the subjects returns to his standing position—see Fig 1). Other indications as in Fig 3.

Most of the *COM* deflection and the associated lower limb joints range of motion take place during *land*_1_ (Fig 5). During this phase, the increase of *F*_z_ is associated with a decrease of *S*_z_ and the graph of *F*_z_ *vs S*_z_ presents a linear relation. The slope of this relation represents the overall leg-spring stiffness generated by the lower limb muscles. At 1 *g*, the mass-specific stiffness *k*_1_ = 181±70 s^-2^ and *k*_1_ increases with increasing gravity (Fig 6). This increase is more pronounced in *A300* than in *SLS-*condition.

During and after *land*_2_ (*i*.*e*. until the end of the *CMJ*), the mass-specific overall stiffness (*k*_2_) and damping coefficient (*c*_2_) of the damped harmonic oscillator system are 43±58 s^-2^ and 9±4 s^-1^, respectively (Fig 6). During this period, there is no significant effect of gravity on the stiffness (*k*_2_). In contrast, the damping coefficient (*c*_2_) increases with augmented gravity. Both *k*_2_ and *c*_2_ are similar in the *A300*- and in the *SLS*-conditions (Fig 6).

## Discussion

The purpose of this study is to analyze the motor control of landing after a *CMJ* at different hypergravity level. Additionally, hypergravity was simulated in two ways, altering the otolithic and proprioceptive inputs differently. Our results show that participants are able to jump and control landing in hypergravity up to 1.6 *g* both in the *A300-* and in the *SLS-*conditions, however they modify their landing strategy differently, according to the experimental condition.

### Landing without rebound

Subjects were instructed to land from a jump without rebounding on the ground. In other words, after *TD*, both feet had to stay in contact with ground until the end of the movement. This behavior affects the biomechanics of landing; indeed, when bouncing on the ground, the energy lost during the deceleration phase after *TD* can be partly stored as elastic energy in the muscle-tendon units of the lower limbs, to be restored during the acceleration phase preceding take-off [24]. In this case, muscles are generating an overall constant leg-spring stiffness during contact [5].

On the contrary, when landing from a jump without rebounding, the mechanical energy accumulated during the falling phase of the jump must be dissipated after *TD*. Newman et al. [25] proposed a damped harmonic oscillator model from touchdown until the end of the landing phase, assuming a constant stiffness, *k* and a constant damping coefficient, *c*. However, the model of Newman et al. [25] does not fit closely to our experimental data neither at 1 *g* (*r* ^2^ = 0.64±15, *k* = 55.8±66.3 s^-2^ and *c* = 5.2±2.3 s^-1^, *n* = 48), nor in hypergravity (*r* ^2^ = 0.46±0.18, *n* = 278). In contrast, Dyhre-Poulsen et al. [6] suggested that muscles change the overall mechanical properties of the lower limb during landing from a spring to a damper. Our model illustrates this idea. Here, we divide the landing in two distinct phases. First, the loading phase (*land*_1_) during which the lower limb muscles act like a stiff spring to absorb the impact with the ground. During this phase, only a small amount of energy is dissipated since the velocity of the *COM* changes little (see Figs 1 & 2). Second, the rest of the landing phase during which muscles act like a spring-damper unit to dissipate the energy of the jump. The strong correlation between the experimental and computed force-time curves observed during these two phases corroborates the idea of Dyhre-Poulsen et al. [6] that muscles are modulating their mechanical properties throughout landing.

### Effect of gravity level

When gravity increases, the modifications of the landing strategy present the same trend both in the *A300-* and in *SLS-*conditions. These changes occur mainly during pre-landing and *land*_1_. With increasing gravity, the lower limb joints are more flexed at *TD* (Figs 2 & 4), most likely due to a greater activity of *TA* and *BF* muscles during the aerial phase (Fig 4). According to McKinley and Pedotti [26], an increased dorsiflexion of the ankle at *TD* may contribute to stabilize landing by decreasing the time during which the sole of the foot is not fully in contact with the ground. We observe that with increased gravity, subjects adopted a strategy that could enhance the stability during landing, *i*.*e*. a greater dorsiflexion of the ankle at *TD* and a shorter *land*_1_ duration (Fig 5 and Table 3). In addition, the greater knee flexion at *TD* may reduce the risk of anterior cruciate ligament injury, as suggested by Dai et al. [27] and Decker et al. [28].

At *TD*, the forefeet touch the ground, inducing dorsiflexion of the ankle until heel-contact, which corresponds to *F*_z-peak_ [7, 13, 29, 30]. When gravity increases, the range of motion (*RoM*_1_ in Fig 5) is decreased and the maximal vertical force (*F*_z-peak_ in Fig 3) is increased. This greater force with a smaller displacement is obtained by increasing the overall leg-spring stiffness (*k*_1_ in Fig 6), which may prevent the joints to collapse during the loading phase. Several authors have reported that the increase in the leg-spring stiffness is mainly induced by an increased dorsiflexion of the ankle at *TD*, both in hopping [31] and in single leg landings [32]. An increase in *k*_1_ with an increased dorsiflexion of the ankle at *TD* is also observed in our study at all gravity levels.

### *A300- vs SLS*-gravity simulation

Our results show that subjects modify their landing strategy with hypergravity in both experimental conditions. However, these modifications are enhanced in *A300* as compared to *SLS*. Indeed, in *A300*, an enhanced 'gravitational field' is applied to the whole body, limb-segments feel heavier and the otolithic system is submitted to a constant additional acceleration both in static and dynamic situations. On the contrary, in *SLS*, an additional force is only applied to the trunk, the weight of the limb-segments is unaltered and the otolithic system only feels a greater acceleration during dynamic situations.

To our knowledge, the landing strategies in hypergravity have only been studied in drop jumps and repetitive hops, using a Subject Loading System, with gravity levels of maximum 1.2–1.3 *g* [20, 21]. When gravity increases, these authors reported small differences in the timing and the amplitude of the pre-landing muscular activity, which are in agreement with our observations both in the *SLS-* and *A300*-conditions (Fig 4). Meanwhile our observations show that in the *A300*-condition, the post-landing *EMG* activity is more important than in the *SLS*-conditions (Fig 4).

Pre-landing *EMG* activity and early *EMG* responses after *TD* are pre-programmed relative to the expected instant of *TD* [33, 34]. This suggests that the modifications observed during the pre-landing phase and during *land*_1_ are likely due to centrally generated motor command based on sensory information [35]. When hypergravity is applied on the whole body, participants modify their motor command in such a way that the joints are more flexed at *TD* and that the post-landing *EMG* activity is enhanced. This behavior could be due, at least in part, to perceptual errors because of the unusual otolithic signals and/or to differences in the vertical velocity of the *COM* at touchdown (see Methodological Limitations).

The difference in behavior between *A300-* and *SLS-*conditions could also be due to an alteration of the visual system during the *A300-*sessions. Indeed, it has been reported that in a centrifuged room the otolith organs induces changes in the oculomotor control of pointing tasks, resulting in an apparent rise of objects [36]. This so-called 'elevator illusion' could also affect the motor control of landing in the *A300*-condition.

### Methodological limitations

Both vertical velocity of the *COM* at *TD* and simulated gravity level were statistically different between *A300-* and *SLS-*sessions (Table 3), but average differences were small: less than 0.18 m.s^-1^ (Fig 3) and within 0.02 *g* (see Methods), respectively. Santello et al. [10] reported an effect of the height of fall, and thus of the vertical velocity of the *COM* at *TD*, on the control of landing. More specifically in landings at 1 *g* from 0.2 m and from 0.4 m high boxes (corresponding to vertical velocity of the *COM* at *TD* of respectively 2.0 and 2.8 m.s^-1^), *F*_z-peak_ was 1.9 and 2.5 times body weight, respectively. As a result in their study, a 0.8 m.s^-1^ difference led to a 0.6-times *BW* difference in *F*_z-peak_. In the current study, the maximal difference of 0.17 m.s^-1^ between 1.2 *g-A300* and 1.2 *g-SLS* led to a difference of 1-times *BW* (Fig 3). Thus, we suggest that the differences in *F*_z-peak_ are only marginally influenced by different vertical velocity of the *COM* at *TD* and likely more related to changes in landing technique (Fig 5) between *A300-* and *SLS-*sessions.

As specified in the Methods, gravity levels were not randomized in the *A300* for security reasons, and were reproduced similarly with the *SLS*. We acknowledge that this choice of an incremental administration of the gravity levels may have influenced the adaptation strategies to the gravity constraints. However, to avoid a carry-over effect from the previous gravity level to the next in this current incremental order, the 3 first trials of each gravity level were discarded.

No specific instruction about the landing technique was given to the subjects. This may explain why the landing strategy slightly differs from one subject to another (*e*.*g*. the important standard deviations observed in Figs 4 & 6 during landing on Earth at 1 *g*). Despite differences in the landing technique among participants, the within-subject ANOVA revealed highly significant differences with increased gravity level, gravitational environment changes and interaction between gravity level and gravitational environment (Table 2). This suggests that our subjects modified similarly their landing strategy according to the experimental conditions.

## Conclusion

Even if in daily living activities, gravity can be perceived as a constant factor, the *CNS* is able to modify the motor control of landing in an 'enhanced gravity field'. Indeed, subjects are able to jump and land in a hypergravity environment up to 1.6 *g*. Both in 1 *g* and in hypergravity, the overall properties of the lower limb muscles change throughout landing from a stiff spring to a compliant spring-damper. With increasing gravity, changes in the landing strategy are mainly observed during the pre-landing phase and during the loading phase (*land*_1_). Since pre-landing and early *EMG* responses are pre-programmed, our results suggest that these modifications are most likely due to a change in the central command, based on sensory information. These modifications are more important in hypergravity simulated by a centrifugal force in an airplane (*A300*) than in hypergravity simulated by a pull-down force in the laboratory (*SLS*), most likely because the proprioceptive and otolithic signals are more altered in the *A300*.

## Supporting Information

S1 Dataset Trial by trial data. (XLSX)
